# Current advances and challenges in Managing Hereditary Diffuse Gastric Cancer (HDGC): a narrative review

**DOI:** 10.1186/s13053-024-00293-5

**Published:** 2024-10-08

**Authors:** L. van der Sluis, J.M. van Dieren, R.S. van der Post, T.M. Bisseling

**Affiliations:** 1 Department of Gastroenterology, Radboud university medical centre, Nijmegen, The Netherlands; 2 Department of Gastrointestinal Oncology, The Netherlands Cancer Institute, Amsterdam, The Netherlands; 3 Department of Pathology, Radboud university medical centre, Nijmegen, The Netherlands

**Keywords:** HDGC, *CDH1*, *CTNNA1*, Diffuse gastric cancer, Endoscopic surveillance

## Abstract

More than 25 years ago, *CDH1* pathogenic variants (PVs) were identified as the primary cause of hereditary diffuse gastric cancer (HDGC), an inherited cancer syndrome that increases the lifetime risk of developing diffuse gastric cancer (DGC) and lobular breast cancer (LBC). Since DGC is associated with a poor prognosis, a prophylactic total gastrectomy (PTG) is currently the gold standard for reducing the risk of DGC in *CDH1* PV carriers. However, as germline genetic testing becomes more widespread, many *CDH1* PV carriers have been identified, including in families with lower penetrance levels or without a history of gastric cancer (GC). When including these families, recent findings suggest that the cumulative lifetime risk of developing advanced DGC is much lower than previously thought and is now estimated to be 13–19%. This lower risk, combined with the fact that around one third of the *CDH1* PV carriers decline PTG due to potential lifelong physical and psychological consequences, raises critical questions about the current uniformity in recommending PTG to all *CDH1* PV carriers. As a result, there is a growing need to consider alternative strategies, such as endoscopic surveillance. However, despite the currently lower estimated risk of infiltrative (advanced) DGC, almost every PTG specimen shows the presence of small low-stage (pT1a) signet ring cell (SRC) lesions of which the behaviour is unpredictable but often are considered indolent or premalignant stages of DGC. Therefore, the primary goal of surveillance should be to identify atypical, deeper infiltrating lesions rather than every SRC lesion. Understanding the progression from indolent to more infiltrative lesions, and recognizing their endoscopic and histological features, is crucial in deciding the most suitable management option for each individual.

## Background

Adenocarcinoma, the predominant type of gastric cancer (90–95%), is histologically subdivided into intestinal and diffuse subtypes [1]. The intestinal subtype exhibits glandular or intestinal architecture, while the diffuse subtype demonstrates signet ring cells (SRCs) or poorly cohesive cells diffusely infiltrating the gastric wall. The intestinal subtype (55–70%) is more common in older men and linked to environmental factors like *Helicobacter Pylori* infection, whereas the diffuse subtype (30–44%), which is associated with familial occurrence, is more common in younger women [2–4]. Familial occurrence accounts for 5–10% of all gastric cancers, with only 1–3% attributed to hereditary cancer syndromes with a known gene mutation [4, 5]. Among these, hereditary diffuse gastric cancer (HDGC) is an autosomal dominant syndrome that predisposes individuals to diffuse gastric cancer (DGC) and lobular breast cancer (LBC). HDGC is primarily caused by a pathogenic variant or likely pathogenic variant (PV/LPV) in the *CDH1* gene, with an incidence of 5 per 100.000 individuals, and less commonly by PV/LPV in the *CTNNA1* gene [6].

The prognosis for DGC remains poor and relatively unchanged due to its aggressive behaviour, late-stage diagnosis, and worse response to treatment compared to the intestinal type GC [3]. This and the uncertainty surrounding safe endoscopic surveillance options has led to recommendation for prophylactic total gastrectomy (PTG) for individuals with *CDH1* PV [3, 7]. However, this approach needs to be reconsidered as recent studies suggest the risk of developing advanced DGC is lower than initally thought and depends on the penetration in the family [8]. Also, *CTNNA1* PV carriers are probably at a lower risk than *CDH1* PV carriers. The reported lifetime risk for developing LBC ranges between 37% and 55% for individuals with *CDH1* PV, depending on the selection criteria for the study population [8–10]. The management of lobular breast cancer (LBC) is beyond the scope of this review article.

Current challenges in managing (H)DGC include the increasing identification of carrier families through widespread testing, the variability in penetrance levels even within families, and patient preferences for less invasive management options. These challenges underscore the need to explore endoscopic surveillance, enabled by better understanding of early DGC detection and greater expertise among medical professionals, including gastroenterologists and pathologists. This review summarizes current knowledge on HDGC, focusing on its clinical management, advancements in insights, and ongoing challenges in endoscopic surveillance.

### Genetics and molecular mechanisms

HDGC is primarily caused by a PV/LPV in the *CDH1* gene, encoding the adhesion protein E-cadherin [11]. Guilford first discovered the association between E-cadherin mutations and DGC in three New Zealand’s Māori families in 1998 [12, 13]. E-cadherin’s role in cell-cell adhesion is well-documented [14]. Recent work using human *CDH1* knock-out organoid models shows that E-cadherin deficiency leads to displacement of dividing cells by disruption of the spindle orientation, providing new insights into DGC development [15]. *CDH1* PVs can be categorized into different mutation subtypes, with nonsense mutations and deletions being associated with the highest risk of developing GC [16]. The incidence of *CDH1* PVs varies across geographical areas, with the highest percentages found in European individuals and in New Zealand, both of which are considered as low-risk GC areas [17, 18]. However, within Europe, Belarus, Portugal and Italy represent relevant areas for GC prevalence. This could at least be partly explained by distinct healthcare resources, screening programs and the consistent presence of the Māori ethnicity within the New Zealand population.

In less than 2% of HDGC-families, a *CTNNA1* PV is identified, encoding α-E-catenin. This protein indirectly binds to E-Cadherin through β-catenin, forming the cadherin/catenin complex essential for regulating cell-cell adhesion via the actin cytoskeleton [19, 20]. Other hereditary syndromes caused by mutations in mismatch repair (MMR) genes (*MLH1*/*MSH2*/*MSH6*/*PMS2*) or homologous recombination deficiency (HRD) genes (*BRCA1*/*BRCA2*/*PALB2*/*RAD51C*) can also explain some familial gastric cancer cases [21, 22]. A substantial part of families with GC aggregation and/or early-onset GC lacks an identified germline causative gene. Recent studies have identified the *RHOA* gene and *CTNND1* gene as possible causative genes, both involved in cell adhesion and signal transduction [23, 24]. *RHOA* functions via GTPase activity, while *CTNND1* encodes protein product catenin delta-1 (p120ctn), which directly interacts with E-Cadherin.

### Clinical presentation and penetrance

*CDH1* is a pleotropic gene, meaning that mutations can lead to a plethora of effects [25]. Besides DGC and LBC, *CDH1* PVs are also associated with blepharocheilodontic syndrome (BCD), characterized by eyelid malformations, cleft lip/palate (CL/P), and dental anomalies, as well as non-syndromic CL/P cases [26, 27]. Besides DGC, *CTNNA1* PVs are linked to macular dystrophy [28, 29]. The exact risk of LBC in *CTNNA1* PVs has not been clarified yet, but the prevalence of LBC is not increased in carriers identified via multigene panel testing compared to individuals with negative multigene panel testing (unpublished data, Herrera-Mullar et al.).

After the discovery of the *CDH1* gene, early lifetime risk estimates for developing DGC were reported to be as high as 80% [30]. However, there early studies were influenced by ascertainment bias due to an enrichment for highly affected families. In recent years, risk estimates have been adjusted lower, with U.S. cohorts not pre-selected based on clinical HDGC criteria reporting risks of 37–42% for men and 22–33% for women [9, 10]. Moreover, a recent American study suggests even lower risks of 7–10% irrespective of family history, although this study also confirms that risks can increase up to 38% when there is a greater DGC penetration in the family [8]. The median age at DGC diagnosis is 39 years for women and 44 years for men [31]. The lifetime risk of LBC for women with *CDH1* mutations ranges from 37% to 55%, depending on the study population [8–10].

The risk of GC in carriers of the *CTNNA1* PV is less well defined. A first estimate suggested a cumulative risk of 49–57% by age 80 [32], but this was a study in families that were highly enriched with DGC cases. A more recent multigene panel testing study from the U.S. indicate a significantly lower prevalence of overall GC (all pathologies) in *CTNNA1* PV carriers compared to *CDH1* PV carriers (unpublished data, Herrera-Mullar et al.). Specifically, this study found that 2.6% of *CTNNA1* PV heterozygotes reported GC and 1.1% DGC specifically, compared to 16% of *CDH1* PV carriers.

To identify patients for genetic testing of *CDH1* and/or *CTNNA1*, the International Gastric Cancer Linkage Consortium (IGCLC) has established criteria that are updated every 5 years. These criteria include early-onset and/or clustering of DGC and/or LBC, Māori ethnicity, and the presence of CL/P [6, 33]. In a series from the U.S. population, *CDH1* PV carriers presented with mixed gastric/breast cancer in 36%, breast cancer only in 36%, gastric cancer only in 16%, or no cancers in 12% of families, with only 46% meeting the 2015 HDGC criteria [9]. In contrast, a European cohort of 176 *CDH1* PV/LPV carrier families showed a different phenotype distribution: 52% had gastric cancer only, 38% had both breast and gastric cancer and 9% had breast cancer only [11]. In this study 84% fulfilled the 2015 or 2020 HDGC criteria. These differences are primarily due to detection bias, resulting from varying approaches to genetic testing across different regions.

### Management of DGC and surveillance endoscopy

Patients with HDGC and *CDH1* PV are advised to consider prophylactic total gastrectomy (PTG) due to its effectiveness in reducing gastric cancer risk [6]. However, around 30% of *CDH1* PV carriers decline PTG due to concerns about their age, fertility, positive surveillance beliefs, and negative family experiences [34, 35]. PTG carries significant post-operative risks including anastomotic leaks and strictures, adhesions, jejunostomy-related complications, infections and hernias [36, 37]. Long term physical sequelae of living without stomach include significant mean weight loss of 15–23%, chronic abdominal discomfort, and dumping syndrome [34, 37, 38]. Psychosocial impact includes negative body image, identity issues, anxiety disorder, bipolar disorder and depression [37, 39, 40]. Laszkowska et al. (2020) calculated optimal PTG ages as 39 for men and 30 for women based on quality-adjusted life years (QALYs), which is older than recommended according to the IGCLC guidelines [41].

For patients who decline or postpone PTG, annual endoscopic surveillance in an expert centre is recommended [6]. Surveillance is also advised for asymptomatic *CTNNA1* PV carriers, HDGC-like individuals, and those with *CDH1* variants of unknown significance (VUS) or no family history of DGC. The current IGCLC guideline recommends performing both targeted and 28–30 random biopsies during surveillance [6]. Detection rates of early pT1a signet ring cell carcinoma (SRCC) using gastroscopy show wide variation between studies, ranging from 20 to 60%, even when adhering to the ‘guideline protocol’ or the Bethesda protocol, which includes 80 random biopsies [42–45]. Theoretical models indicate that around 1800 random biopsies are needed to capture at least 1 cancer focus and maintain a 90% detection rate, which is not feasible in a clinical setting [46]. Additionally, repeated biopsy can lead to scar formation, mimicking SRC lesions [47]. Given the challenges, the effectiveness of surveillance protocols must be improved. A retrospective Dutch series found that SRCC lesions were identified in 69% of patients via endoscopic surveillance, mainly through targeted biopsies. Targeted biopsies had a significant higher yield (11%) compared to random biopsies (0.9%), suggesting that the value of random biopsies is questionable. Notably, no advanced tumours were missed endoscopically during follow-up, and all were recognised at baseline endoscopy [48]. Another study in a U.S. cohort of *CDH1* PV individuals showed that development of progressing lesions (*≥* T1b) was infrequent in individuals undergoing surveillance [49]. Lee et al. developed a diagnostic framework with three endoscopic criteria to detect early T1a SRCC lesions and to distinguish them from other gastric abnormalities, achieving 67% sensitivity, with only 4% of the non-suspicious lesions showing SRCC histology [50].

**Fig. 1:**
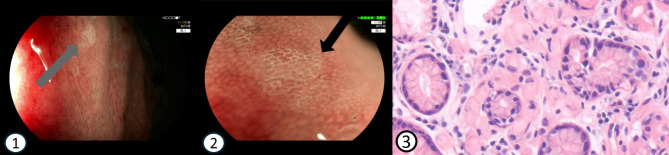
Endoscopic and histopathological images of early HDGC gastric lesions. 1-2: Endoscopic visible T1a lesion in 25-year-old female (*Fuijnon*)1) Blue Light Imaging (BLI), normal magnification (grey arrow). 2) BLI, zoom *1:100 x 1.15*, irregular microsurface pit pattern (black arrow). 3: Histology of mucosal signet ring cell carcinoma

These findings together suggest that annual endoscopic surveillance at an expert centre could serve as an alternative to PTG in *CDH1* mutation carriers, pointing out the importance of gastroscopic examination with targeted biopsies and the development of a diagnostic framework. Such a framework should focus on recognizing atypical infiltrating SRCC lesions while minimizing scar formation, rather than to identify every single pT1a SRCC lesion [51, 52]. This approach is especially important because many SRCC lesions likely display indolent behaviour [53]. Supporting this, SRCC foci are found in over 95% of PTG specimens [54]. Even in PTG specimens from *CDH1* PV individuals without family history of DGC, these lesions seem to be abundantly present [36]. Nevertheless, 60–90% of these individuals are unlikely to develop advanced DGC [8, 36, 43, 55]. To differentiate between indolent early T1a lesions and atypical more infiltrative lesions (atypical T1a, or *≥* T1b), we have proposed a three-tier classification system as diagnostic framework, combining endoscopic and histologic characteristics, each with different clinical implications [52]. T1a SRCC is characterized endoscopically as a flat, pale lesion with an irregular microvascular pit pattern, which can be better visualized using Blue Light Imaging or Narrow Band Imaging endoscopy (Fig. 1, panels 1–2). Histology confirms the diagnosis of SRCC (Fig. 1, panel 3). Endoscopic signs of deeper infiltrating lesions (atypical T1a, or *≥* T1b) may include thickening of gastric folds, elevation or depression, changed vascular pattern and a coarse pit pattern. This is the first diagnostic framework specifically focused on recognizing signs of progression and needs broader validation to assess its applicability and reliability in clinical practice.

### Future perspectives in alternatives for PTG

To date, there have been no effective systemic treatment strategies specifically for HDGC. However, recent research has shown promising results for future management options. Preclinical studies using E-cadherin-deficient cells and gastric and mammary gland organoids have demonstrated susceptibility to specific multikinase inhibitors and histone deacetylase (HDAC) inhibitors. These drugs exhibit anticancer effects by promoting apoptosis and maintaining the integrity of the epithelial plane [56]. Additionally, combining HDAC inhibitors with statins has been shown to synergistically inhibit cell survival in breast cell lines lacking functional *CDH1* [57]. There is also evidence supporting immune-mediated control of early-stage tumour growth in HDGC, suggesting another potential target for pharmacological prophylaxis or treatment [58].

Epidermal Growth Factor Receptor (EGFR) plays a crucial role in cellular growth, survival, and proliferation by activating multiple downstream pathways, including PI3K-AKT, PI3K-mTOR, c-Src, FAK, TOPO2-related and RAS-RAF-MEK-ERK [59]. *CDH1* mutant cells are less able to suppress EGFR activation, which may explain their selective sensitivity to targeted inhibition of EGFR effectors such as PI3K, mTOR, MEK, c-Src, FAK and TOPO2.

Finally, inhibitors of sphingolipid metabolism (e.g. PF-543), endocytosis (PP1, PP2, SU6656, chlorpromazine), vesicle formation (MNS), and autophagy (chloroquine, hydro-chloroquine) have been identified as synthetic lethal in *CDH1* mutant breast cell lines and/or organoid models of HDGC [60]. These drugs offer potential new strategies for preventing HDGC-related malignancies as an alternative to PTG, but clinical trials are needed to prove their effectiveness and safety.

## Conclusion

Managing HDGC presents significant challenges, given the variable penetrance of *CDH1* PVs among individuals, the limitations of surveillance methods, and the profound impact of PTG as the standard of care. Addressing these challenges is crucial for optimizing care in HDGC patients. Future research should focus on development of personalized management plans and refinement of endoscopy surveillance protocols that fits the goal of surveillance, especially in recognizing signs of progression and deeper infiltrating lesions. Longer follow-up studies are needed to demonstrate the safety of annual endoscopic surveillance. Additionally, there is a critical need for better prediction of the behaviour of SRC lesions and the timeline for progression to stage T2 disease. Until then, we must weigh the decision to undergo PTG in every patient, providing in-depth information about the advantages and disadvantages of PTG and endoscopic surveillance risks.

## Data Availability

No datasets were generated or analysed during the current study.

## Abbreviations

HDGC: Hereditary Diffuse Gastric Cancer
PV: Pathogenic variants
PTG: Prophylactic total gastrectomy
GC: Gastric cancer
SRC: Signet ring cell
LBC: Lobular breast cancer
LPV: Likely pathogenic variant
DGC: Diffuse gastric cancer
MMR: Mutations in mismatch
HRD: Homologous recombination deficiency
BCD: With blepharocheilodontic syndrome
CL/P: Cleft lip/palate
QALY’s: Quality-adjusted life years
VUS: Variants of unknown significance
SRCC: Signet ring cell carcinoma
BLI: Blue light imaging
HDAC: Histone deacetylase
